# Supplementary material for: Unveiling the Bioactive Potential of the Invasive Jellyfish Phyllorhiza punctata Through Integrative Transcriptomic and Proteomic Analyses
Source: Biomolecules. 2025 Aug 4;15(8):1121. doi: 10.3390/biom15081121 (PMC12383608; doi:10.3390/biom15081121)
Supplement: Supplementary file 1 [file biomolecules-15-01121-s001.zip › Suplementary Material/FileS3/PEPFOLD4/XP_065066891_9060/clusters.html]

 PEP-FOLD clusters


Download archive of all models  here   

|  |  |  |
| --- | --- | --- |
| # | file | sOPEP |
| # cluster 1 - Size: 74 |  |  |
|  | XP\_065066891\_9060-model1 | -18.7676 |
|  | XP\_065066891\_9060-model1.1 | -18.7423 |
|  | XP\_065066891\_9060-model1.2 | -18.7423 |
|  | XP\_065066891\_9060-model1.3 | -18.7377 |
|  | XP\_065066891\_9060-model1.4 | -18.7223 |
|  | XP\_065066891\_9060-model1.5 | -18.7165 |
|  | XP\_065066891\_9060-model1.6 | -18.65 |
|  | XP\_065066891\_9060-model1.7 | -18.65 |
|  | XP\_065066891\_9060-model1.8 | -18.6408 |
|  | XP\_065066891\_9060-model1.9 | -18.6408 |
|  | XP\_065066891\_9060-model1.10 | -18.6408 |
|  | XP\_065066891\_9060-model1.11 | -18.6408 |
|  | XP\_065066891\_9060-model1.12 | -18.6408 |
|  | XP\_065066891\_9060-model1.13 | -18.6408 |
|  | XP\_065066891\_9060-model1.14 | -18.6408 |
|  | XP\_065066891\_9060-model1.15 | -18.6408 |
|  | XP\_065066891\_9060-model1.16 | -18.6408 |
|  | XP\_065066891\_9060-model1.17 | -18.6315 |
|  | XP\_065066891\_9060-model1.18 | -18.6315 |
|  | XP\_065066891\_9060-model1.19 | -18.6315 |
|  | XP\_065066891\_9060-model1.20 | -18.6315 |
|  | XP\_065066891\_9060-model1.21 | -18.6315 |
|  | XP\_065066891\_9060-model1.22 | -18.6315 |
|  | XP\_065066891\_9060-model1.23 | -18.6315 |
|  | XP\_065066891\_9060-model1.24 | -18.6315 |
|  | XP\_065066891\_9060-model1.25 | -18.619 |
|  | XP\_065066891\_9060-model1.26 | -18.619 |
|  | XP\_065066891\_9060-model1.27 | -18.619 |
|  | XP\_065066891\_9060-model1.28 | -18.619 |
|  | XP\_065066891\_9060-model1.29 | -18.619 |
|  | XP\_065066891\_9060-model1.30 | -18.619 |
|  | XP\_065066891\_9060-model1.31 | -18.619 |
|  | XP\_065066891\_9060-model1.32 | -18.619 |
|  | XP\_065066891\_9060-model1.33 | -18.619 |
|  | XP\_065066891\_9060-model1.34 | -18.6158 |
|  | XP\_065066891\_9060-model1.35 | -18.6123 |
|  | XP\_065066891\_9060-model1.36 | -18.6033 |
|  | XP\_065066891\_9060-model1.37 | -18.6033 |
|  | XP\_065066891\_9060-model1.38 | -18.6033 |
|  | XP\_065066891\_9060-model1.39 | -18.6007 |
|  | XP\_065066891\_9060-model1.40 | -18.6007 |
|  | XP\_065066891\_9060-model1.41 | -18.5965 |
|  | XP\_065066891\_9060-model1.42 | -18.5965 |
|  | XP\_065066891\_9060-model1.43 | -18.5965 |
|  | XP\_065066891\_9060-model1.44 | -18.5965 |
|  | XP\_065066891\_9060-model1.45 | -18.5965 |
|  | XP\_065066891\_9060-model1.46 | -18.5859 |
|  | XP\_065066891\_9060-model1.47 | -18.5859 |
|  | XP\_065066891\_9060-model1.48 | -18.5825 |
|  | XP\_065066891\_9060-model1.49 | -18.5825 |
|  | XP\_065066891\_9060-model1.50 | -18.5825 |
|  | XP\_065066891\_9060-model1.51 | -18.5801 |
|  | XP\_065066891\_9060-model1.52 | -18.5801 |
|  | XP\_065066891\_9060-model1.53 | -18.5694 |
|  | XP\_065066891\_9060-model1.54 | -18.5585 |
|  | XP\_065066891\_9060-model1.55 | -18.5559 |
|  | XP\_065066891\_9060-model1.56 | -18.5535 |
|  | XP\_065066891\_9060-model1.57 | -18.5508 |
|  | XP\_065066891\_9060-model1.58 | -18.5321 |
|  | XP\_065066891\_9060-model1.59 | -18.5296 |
|  | XP\_065066891\_9060-model1.60 | -18.5278 |
|  | XP\_065066891\_9060-model1.61 | -18.5278 |
|  | XP\_065066891\_9060-model1.62 | -18.5263 |
|  | XP\_065066891\_9060-model1.63 | -18.5263 |
|  | XP\_065066891\_9060-model1.64 | -18.5236 |
|  | XP\_065066891\_9060-model1.65 | -18.5215 |
|  | XP\_065066891\_9060-model1.66 | -18.4895 |
|  | XP\_065066891\_9060-model1.67 | -18.4778 |
|  | XP\_065066891\_9060-model1.68 | -18.4703 |
|  | XP\_065066891\_9060-model1.69 | -18.4665 |
|  | XP\_065066891\_9060-model1.70 | -18.4562 |
|  | XP\_065066891\_9060-model1.71 | -18.4562 |
|  | XP\_065066891\_9060-model1.72 | -18.4252 |
|  | XP\_065066891\_9060-model1.73 | -18.0678 |
| # cluster 2 - Size: 26 |  |  |
|  | XP\_065066891\_9060-model2 | -14.6465 |
|  | XP\_065066891\_9060-model2.1 | -14.6455 |
|  | XP\_065066891\_9060-model2.2 | -14.5973 |
|  | XP\_065066891\_9060-model2.3 | -14.5913 |
|  | XP\_065066891\_9060-model2.4 | -14.5586 |
|  | XP\_065066891\_9060-model2.5 | -14.5287 |
|  | XP\_065066891\_9060-model2.6 | -14.5287 |
|  | XP\_065066891\_9060-model2.7 | -14.5287 |
|  | XP\_065066891\_9060-model2.8 | -14.5287 |
|  | XP\_065066891\_9060-model2.9 | -14.5217 |
|  | XP\_065066891\_9060-model2.10 | -14.5129 |
|  | XP\_065066891\_9060-model2.11 | -14.4972 |
|  | XP\_065066891\_9060-model2.12 | -14.4972 |
|  | XP\_065066891\_9060-model2.13 | -14.4972 |
|  | XP\_065066891\_9060-model2.14 | -14.446 |
|  | XP\_065066891\_9060-model2.15 | -14.2412 |
|  | XP\_065066891\_9060-model2.16 | -14.2412 |
|  | XP\_065066891\_9060-model2.17 | -14.2407 |
|  | XP\_065066891\_9060-model2.18 | -14.189 |
|  | XP\_065066891\_9060-model2.19 | -14.1803 |
|  | XP\_065066891\_9060-model2.20 | -14.1029 |
|  | XP\_065066891\_9060-model2.21 | -14.0967 |
|  | XP\_065066891\_9060-model2.22 | -14.0947 |
|  | XP\_065066891\_9060-model2.23 | -14.029 |
|  | XP\_065066891\_9060-model2.24 | -13.9937 |
|  | XP\_065066891\_9060-model2.25 | -13.9787 |
|  |  |  |
